# Correction for Brennan et al., “Maximizing the potential of high-throughput next-generation sequencing through precise normalization based on read count distribution”

**DOI:** 10.1128/msystems.01288-24

**Published:** 2024-12-13

**Authors:** Caitriona Brennan, Rodolfo A. Salido, Pedro Belda-Ferre, MacKenzie Bryant, Charles Cowart, Maria D. Tiu, Antonio Gonzalez, Daniel McDonald, Caitlin Tribelhorn, Amir Zarrinpar, Rob Knight

## AUTHOR CORRECTION

Volume 8, no. 4, e00006-23, 2023, https://doi.org/10.1128/msystems.00006-23. Page 1, notes: “The authors declare no conflict of interest” should read as follows. “Rob Knight is a scientific advisory board member and consultant for BiomeSense, Inc., has equity, and receives income. He is a scientific advisory board member and has equity in GenCirq. He is a consultant and scientific advisory board member for DayTwo and receives income. He has equity in and acts as a consultant for Cybele. He is a co-founder of Biota, Inc., and has equity. He is a cofounder of Micronoma, has equity, and is a scientific advisory board member. The terms of these arrangements have been reviewed and approved by the University of California, San Diego, in accordance with its conflict-of-interest policies. Daniel McDonald is a consultant for BiomeSense, Inc., has equity, and receives income. The terms of these arrangements have been reviewed and approved by the University of California, San Diego, in accordance with its conflict-of-interest policies."

This correction does not change the results or the conclusions of this work.

