# Supplementary figures and images for: Maximizing the potential of high-throughput next-generation sequencing through precise normalization based on read count distribution
Source: mSystems. 2023 Jun 23;8(4):e00006-23. doi: 10.1128/msystems.00006-23 (PMC10469589; doi:10.1128/msystems.00006-23)

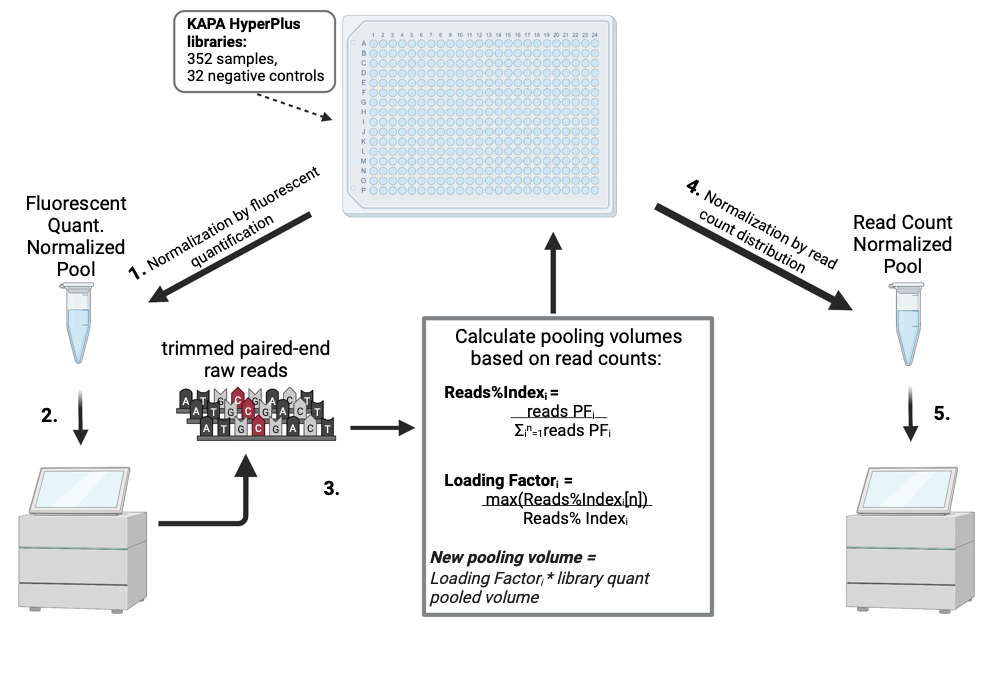

Supplement: Figure S1 — Flowchart of experimental design. 1. KAPA HyperPlus shotgun libraries are quantified using the PicoGreen fluorescence assay (ThermoFisher, Inc) and pooled to approximately equimolar fractions. 2. Pool is sequenced on Illumina's iSeq. 3. The resulting raw reads Passing Filter (PF) is used to calculate a Loading Factor for each library, which is the ratio between the index representing the highest proportion of the total reads PF and the index of each library’s proportion of total reads PF (Illumina. [Internet]. 2019. Available from: https://www.illumina.com/content/dam/illumina-marketing/documents/systems/iseq/single-cell-library-qc-app-note-770-2019-029.PDF). This in turn scales the fluorescent quantified pooled volumes to calculate new pooling volumes. The new pooling volumes are clipped within a reasonable range for acoustic droplet ejection (typically between the range of 10 nL and 1,000 nL, using the Labcyte Echo 550). 4. Libraries are pooled using new pooling volumes. 5. The resulting read count normalized pool is sequenced on illumina's iSeq. Created with BioRender.com. [file msystems.00006-23-s0001.tif]

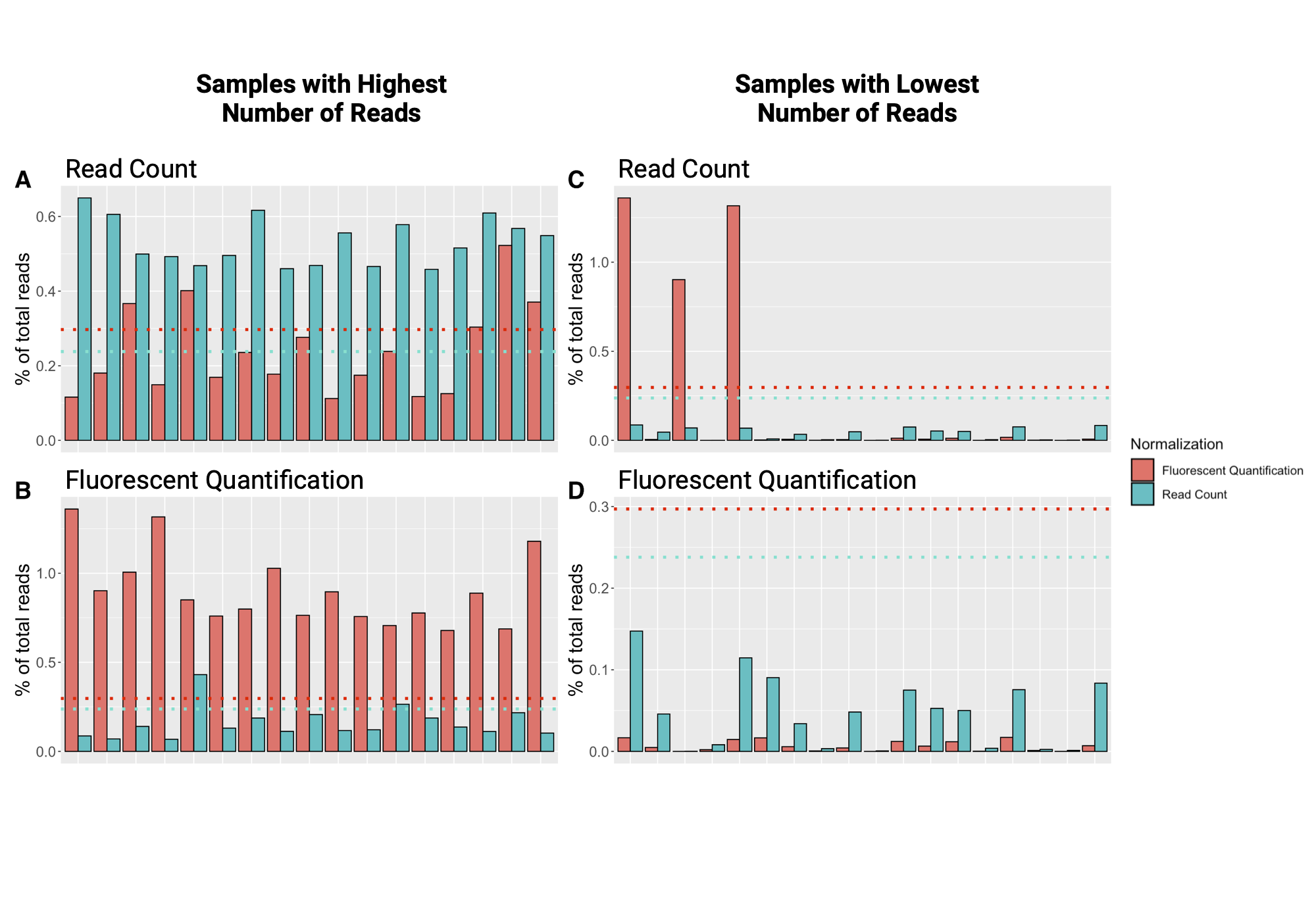

Supplement: Figure S2 — Comparison of the top 5% of samples with the least and most reads for each normalization method. Dotted line represents the median percentage of total reads for both methods (Read Count normalized in turquoise and Fluorescent Quantification normalized in pink/red). (A) Top 5% of samples with the most amount of reads when Read Count normalization was applied. (B) Top 5% of samples with the most amount of reads when Fluorescent Quantification normalization was applied. (C) Top 5% of samples with the least amount of reads when Read Count normalization was applied. (D) Top 5% of samples with the least amount of reads when Fluorescent Quantification normalization was applied. [file msystems.00006-23-s0002.tif]

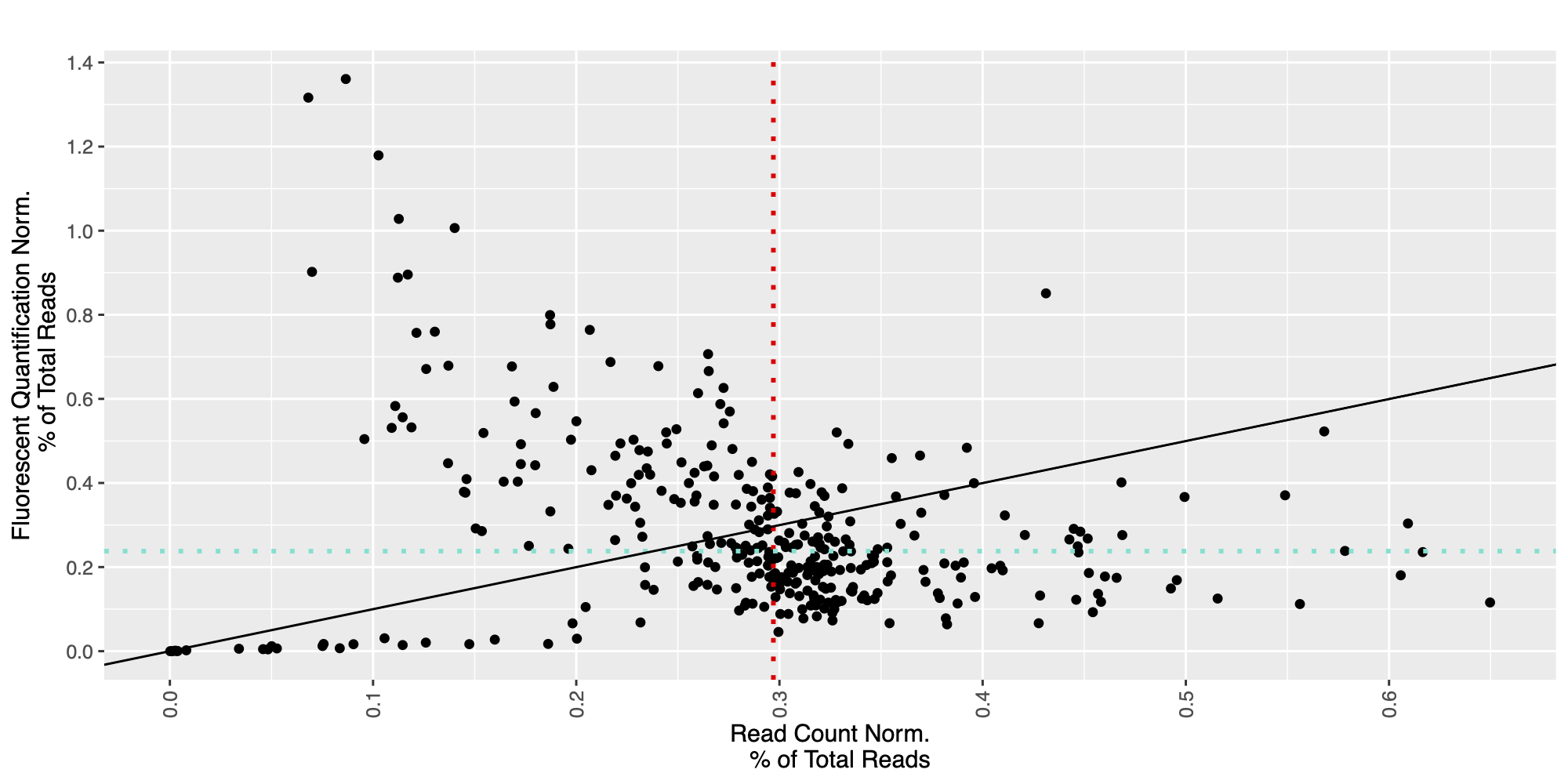

Supplement: Figure S3 — Correlation between proportion of read counts per sample. Dotted lines represent the medians of each method. The diagonal line has a slope of 1. Some overrepresented samples from the Fluorescent Quantification Normalization method [% Total Reads {greater than or equal to} 3X (median % Total Reads )] were over penalized for the subsequent Read Count Normalization method, which resulted in a lower percentage of Total Reads than the median across samples within this normalization method. Nonetheless, the distribution of percentages of Total Reads across samples of the Read Count Normalization method was tighter and the median percentage of Total Reads across samples was higher. [file msystems.00006-23-s0003.tif]
