# Supplementary material for: Maximizing the potential of high-throughput next-generation sequencing through precise normalization based on read count distribution
Source: mSystems. 2023 Jun 23;8(4):e00006-23. doi: 10.1128/msystems.00006-23 (PMC10469589; doi:10.1128/msystems.00006-23)
Supplement: Text S1 — Supplemental Materials and Methods. [file msystems.00006-23-s0004.docx]

TEXT S1

Materials and Methods

Sample Collection and DNA Extraction

Mouse fecal pellets were obtained under the approved Institutional Animal Care and Use Committee (IACUC) protocol from the University of Colorado, number 2539. Nucleic Acids were extracted from 352 samples of mouse fecal pellets using the MagMAX™ Microbiome Ultra Nucleic Acid Isolation Kit and 96-well bead plate (cat. Number A42357, Thermo Fisher Scientific, MA) according to the manufacturer’s instructions. Samples were plated using sterile technique into four 96-well plates with each plate containing 8 blank wells, which served as negative controls.

Fluorescent Quantification for Input Normalization

We quantified the concentration of the resulting gDNA using a miniaturized version of the PicoGreen fluorescence assay (detection window 0.20 - 50 ng/µL) (ThermoFisher, Inc) in 384 well format. We used a 12-point serial dilution (150 ng/µL - 0.156 ng/µL) of λ DNA (Invitrogen, Thermo Fisher Scientific, MA) in 16 replicates as a standard curve and 16 blank wells (containing only PicoGreen master mix) as a negative control. We used Eppendorf’s EPMotion liquid handler to dispense 49 µL of the master mix into wells of black 384 well plates (Corning, NY) (592 reactions). The PicoGreen Master Mix was created as follows: 28,350 µl of Nuclease Free Water, 1,500 µL 20x TE buffer and 150 µL of PicoGreen Dye. We used the SPTLabTech Mosquito to transfer 1 µL of STD and gDNA into their respective wells containing the master mix. Plates were sealed with foil seals, quickly vortexed and centrifuged. The fluorescence of each well was analyzed using a plate reader and the concentration (ng/µL) of each sample was calculated. The Labcyte Echo 550 acoustic liquid handler was used to make dilutions of each sample to ensure equal DNA input into the downstream shotgun library preparation (5 ng at 3.5 µL). If gDNA concentration was not sufficient to reach 5 ng, then a maximum of 3.5 µL was used.

Shotgun Library Preparation

The resulting normalized plate was then used to make shotgun libraries using the KAPA HyperPlus preparation kit (KAPA Biosciences) miniaturized to an approximately 1:10 reagent volume as previously described by Sanders et al. (Sanders JG, Nurk S, Salido RA, Minich J, Xu ZZ, Zhu Q, et al. Genome Biol. 2019 Dec;20(1):226.).

Pooling and Sequencing

The resulting libraries were then quantified using the PicoGreen fluorescence assay as described for the input normalization, with 1µL of library used per reaction. Pooling volumes were then calculated based on the library concentrations (ng/µL) and pooled using the Labcyte Echo 550 acoustic liquid handler to a combined volume of ~130 µL. Samples were pooled within a range of 100 nL – 1000 nL and normalized to 1 ng. The extraction blanks were floored to a volume of 200 nL per blank. The fluorescent quantified normalized pool was then size-selected to 300 - 700 bp using a Pippin Prep electrophoresis instrument (Sage Sciences) and the average fragment length determined by High Sensitivity DNA ScreenTape Analysis (Agilent). This size-selected pool was loaded to Illumina’s iSeq according to the manufacturer's instructions at 90 picomolar (pM) in 20 µL. Adapter trimmed raw reads Passing Filter (*PF*) were used to calculate a Loading Factor, which in turn scales the fluorescent quantified pooled volumes to calculate new pooling volumes. The new pooling volumes were clipped within a reasonable range for acoustic droplet ejection (typically between the range of 10 nL and 1000 nL). Libraries were pooled using these new pooling volumes. The resulting read count normalized pool was size selected, quality controlled, and sequenced on Illumina’s iSeq again according to manufacturer’s instructions. To demonstrate normalization by feature space, we created a third, metatranscriptomics sequencing pool, which was normalized based on the number of reads of interest (non-ribosomal reads) and sequenced on Illumina’s iSeq as mentioned previously. Counts of reads of interest (non-ribosomal reads) were obtained by using SortMeRNA (version v2.1b with default parameters) on adapter trimmed, raw reads PF to partition the metatranscriptomic reads into ribosomal and non-ribosomal reads. The counts of non-ribosomal reads (reads on target, Fig. 2) replaced the raw *reads PF_i_* terms in the numerator and denominator of the Reads%Index calculation (Fig. S1, #3). Processing and analysis code is available on GitHub (<https://github.com/cbrenchy/read_count_normalization.git>).
